# Transmission speed and ratio optimization for heavy-duty electric truck

**DOI:** 10.1016/j.heliyon.2022.e10028

**Published:** 2022-07-21

**Authors:** Elif Gözen, M. Sedat Çevirgen, Emre Özgül

**Affiliations:** Ford Otosan R&D Center, Sancaktepe, 34885, Istanbul, Turkey

**Keywords:** e-Axle, One-dimensional modeling, Heavy-duty, Electric trucks, Transmission, Powertrain optimization

## Abstract

Nowadays, the Heavy-Duty Truck industry is facing one of the most critical questions in the universe. How would it be possible to decrease the CO_2_ emissions? Performing all possible technological steps in conventional engines is only a “part” of the solution. Electrification is a must for both keeping the environment clean and fulfilling the stringent emission regulations successfully. In this paper, the electrification part of the solution is examined. The main goal is to develop a transmission speed and ratio selection methodology that can be used at the early stages of battery electric truck development, especially for conceptual decisions or hardware selection. The authors are focused on the effect of electric axle (e-axle) transmission speed and ratio selection in 27 Tons Battery Electric (BE) Road Truck application. First of all, a one-dimensional vehicle model is created in GT-Suite software. Then, e-axle, electric motor (e-motor) & battery, regenerative braking model block, electric driveline transmission & differential submodels, and the integrated control algorithm are also implemented. In order to complete a thorough analysis, five different transmission designs (2-speed single, 2 + 1 speed split, 2 + 2 speed split, 3-speed single, 4-speed single) with thousands of gear ratio sets are studied. Satisfying the performance requirements with minimum energy consumption in defined Vecto routes is the vital target of these studies. Dual e-motor studies show that the most effective way of decreasing energy consumption is using e-motors at the same speed (up to 5% benefit) and turning off one (up to 0.6% benefit) below specific torque values if the same e-motors are used. Instead of using complex split transmissions, increasing the number of gears in the single transmission is a prominent configuration because it operates the e-motors at a higher efficient point. In the light of these studies, 3-speed single transmission is capable of providing gradeability requirements with the lowest energy consumption and less system complexity.

## 1 Introduction

Reducing CO_2_ emissions is one of the most critical problems that humankind ever encountered. Although emission regulations are getting harder periodically, it is known that further precautions must be taken. Paris Agreement [1] had set a target of keeping global temperature rise under 2 °C (1.5 °C) above the pre-industrial era level. Besides, the World Health Organization’s local air quality limit [2] for human health is 40 *μ*g/m^3^ for NO_*x*_ (20 *μ*g/m^3^ limit is also under discussion.)

Moreover, The Sustainable Europe Investment Plan [3] will include 1 trillion euros of investment over the next decade. The program will support electrification, hydrogen & synthetic fuels from renewable energy sources using carbon capture. Carbon Border Tax will also be introduced to avoid carbon leakage and keep sustainable companies competitive.

Today, the environmental measures are currently in control of the authorized organizations such as the European Union Council. The council has set new emission regulations in May of 2018. According to the regulations, reference fleet-based CO_2_ values must be lowered by 15% by 2025 and 30% by 2030. Vehicle CO_2_ declarations will be simulated by Vecto Tool [4]. Vecto (Vehicle Energy Consumption Calculation Tool) is the new simulation tool developed by the European Commission [5] and shall be used to determine CO2 emissions from Heavy-Duty Vehicles (trucks, buses, and coaches) with a Gross Vehicle Weight above 3500kg. Starting from 1st of January 2019, the tool is mandatory for new trucks under specific vehicle categories in application to the certification legislation under type approval.

New EU Commission President (as of December 2019) Ursula von der Leyen has introduced a “Green Deal” campaign that aims to reduce CO_2_ at least 50% ilo 40% for 2030 in Europe and make Europe the first CO_2_-neutral continent before 2050 [6, 7]. However, it is obvious that 30% CO_2_ consumption reduction cannot be obtained by only developing conventional technologies. To fulfill the requirements, the electrification of the vehicle is a clear obligation. At this point, there are many options to employ, such as mild (48V) hybrid, high-voltage hybrid, battery-electric (BE) trucks, or alternative fuels. The implementation of an electric axle drive (e-axle) is also a strong alternative for further CO_2_ reduction. Achieving the most extended electric drive range with internal combustion engine (ICE) equalized performance is critical for selling electric trucks in the market.

In this study, the authors worked on an electric axle (e-axle) design funded by the EU Horizon 2020 Project. The electric axle is a power source with its own electric motor, battery, transmission, and differential. Ford Otosan is developing an e-axle within the scope of this project as a solution to support the efforts to reduce GHG emissions of heavy-duty trucks. Thanks to its modular structure, e-axle is planned to be used in different truck electrification applications. The project target is implementing an e-axle system in a hybrid truck to gain an 8% CO_2_ emission reduction.

The literature of electrified powertrain transmission design for BE Truck is investigated. Single transmission, split transmission, and planetary gear train transmission designs are the main investigated designs. Although the subject of this study is heavy-duty electric trucks, literature of all vehicle segments is investigated due to the lack of truck studies.

Tian worked on the 2-speed transmission on battery electric vehicle (BEV) application [8]. Powertrain transient responses are observed by a comprehensive and original electrified powertrain system model. Three alternative torque trajectories are proposed to control the motor torque reduction and reinstatement at the first and fifth stages. This study provides a valuable reference for gear shifting control of clutchless automated manual transmission in battery electric vehicles.

Fiori et al. [9] highlights the main advantages of EVs as their ability to recover energy while braking using a regenerative braking system. Vehicle energy consumption models consider an average constant regenerative braking energy efficiency or regenerative braking factors. Their model computes the regenerative braking efficiency by using the instantaneous vehicle operational variables to enhance EV energy consumption models. The impact of auxiliary systems, including the air conditioning and heating systems, on vehicle energy consumption levels are also studied. The results show that the use of the heating and air conditioning system could significantly reduce the EV efficiency and travel range.

The shifting strategy and energy management of a two-motor drive powertrain for extended range electric buses are investigated by Nguyen et al. [10]. It comprises two e-motors connected to interlacing gears of a four-speed automated manual transmission (AMT) through independent input shafts. The total electric consumption is reduced by 8.6% in the two-motor drive powertrain. Therefore, fuel consumption is reduced significantly (by 9.1%) in comparison with the conventional powertrain. The torque fill capability, which improves upshift and downshift quality by eliminating torque interruptions, is a crucial motivation in this study to simulate split transmissions.

Zhang et al. [11] worked on a dual e-motor drive powertrain. New modes switching control methods based on dual-motor centralized and distributed coupling drive systems are studied. This system achieves the centralized and distributed coupling drive and reduces the modes switching shock to improve electric vehicle dynamics performance.

In the study of Zhang et al. [12], two speeds dual-clutch transmission (DCT) and simplified continuously variable transmission (CVT) systems are investigated for BEVs. E-motor efficiency improvement and saved battery energy calculations are conducted from vehicle models built in the Matlab/Simulink environment. Two-speed DCT equipped BEV is reported as a long-term cost effective solution; however, a simplified CVT-equipped BEV can offer a better driving experience, no matter in accelerating, climbing, or shifting.

A detailed study is carried out on a planetary gear system run with two e-motors (sun and ring) by Carlo et al. [13]. This innovative system can operate with higher efficiency than a single e-motor & single transmission configuration in a BEV passenger car. The planetary gear system’s total efficiency improvement is compared with the single transmission driven by a single e-motor.

Battery electric vehicle literature is heavily focused on passenger cars. Since there are limited studies in BEVs, hybrid electric vehicle studies are also examined. Furthermore, the heavy-duty truck based literature is more focused on hybrid options.

Three different hybrid electric powertrain architectures are compared by Xu et al. [14]. Under the economic scenario assumptions, the payback period for hybrid electric heavy-duty trucks is less than six years for the Chinese-World Transient Vehicle Cycle. The hybrid electric powertrain benefit is highlighted as 18% fuel economy improvement when compared to traditional heavy-duty trucks.

In the studies [15, 16], although the usage of multiple e-motors and torque shares are studied for the hybrid electric vehicle (HEV) passenger car, no optimization is made on transmission gear ratios & speeds and the e-motor operation map. In the studies [17, 18], energy & fuel consumption studies are carried out for BEV and HEV vehicles; nevertheless, no detailed study is made on multiple e-motor operations or transmission ratios [19, 20, 21]. studies are focused on hybridization and torque split between e-motor and internal combustion engines or fuel cells.

At the end of the literature survey, authors concluded that most of the studies in the literature were for passenger cars. Obviously, the torque demand of passenger car range is quite low compared with heavy-duty trucks. Hence, higher transmission of power may requires different gear ratios and designs. Furthermore, available studies are evaluating optimization areas individually. Therefore, a new study is required for all possible transmission scenarios (single transmission with multiple speeds and split transmissions) for heavy-duty trucks. Besides, it is critical to combine them with extended torque split and e-motor operation map improvements, which should be scanned in a wide space. The split transmission represents a transmission that could run each e-motor with different speeds and torques.

This paper provides a robust methodology that can be used for the total efficiency optimization of e-axle powertrain for heavy-duty trucks. 27 tons of conventional Road Truck is used and updated to an e-axle driven 27 tons of Battery Electric Road Truck. As a result of the price/performance market research, e-axle is decided to be driven by the same two electric motors [22]. The authors are looking for the simplest and the most cost-effective solution by:

• Running e-motors at the highest total efficiency range,
• Setting algorithm of the torque split between dual e-motors,
• Using different transmission design alternatives (split & single) for dual e-motors,
• Increasing number of alternative transmission speeds,
• Optimizing gear ratio sets per transmission alternatives.

The global optimization of transmission speed and gear ratio is performed to find out the optimum solution and to avoid from converging to a local minima. First, e-truck performance requirements and cycles will be provided in Section 3. Afterward, in Section 2, the one-dimensional e-Truck model is described. Next, alternative transmission configurations are presented in Section 4 with dual e-motor controls. Optimization setup, best transmission ratio sets per configuration variant, and results are provided in Section 5. Finally, the energy consumption comparison of five different transmission configurations, which are 2-speed single, 2 + 1 speed split, 2 + 2 speed split, 3-speed single, and 4-speed single, will be demonstrated.

## 2 One dimensional model of E-truck

Mahmud et al. [21] reported out different simulation tools that can be used for electrification studies. They have examined one hundred and twenty-five different simulation tools that can be utilized for electrification studies. They also reported that one-dimensional tools such as GT-Suite are capable of analyzing hybrid powertrain and engine.

The e-truck model is generated in the GT-Suite platform due to the simplicity and capability of the program. GT-Suite [23] is a CAE simulation software that provides a modeling environment and enables performance analysis for vehicle system simulations.

The one-dimensional truck model calculates tractive wheel torque that is required to reach the target speed at the target grade by using the transient cycle data (speed-time, slope-distance) as input. Depend on system efficiency and gear ratios, speed and torque are transmitted to the e-motor. Based on required tractive torque and speed per second, e-motor efficiency is calculated from the e-motor map. The required e-motor power is demanded from the battery. The battery is simulated with the open circuit voltage (OCV) and internal resistance maps. The consumed electric energy from the battery is calculated instantly.

The prime components of the electric powertrain are modeled in GT-Suite based on component supplier data. Two e-motor drivelines are constituted and connected to the transmission depending on the transmission alternatives. The settled battery size is 240 kWh. Battery power limitation is added in the model to overcome the battery current limit exceeding issues. 400 A battery current charge and discharge limits are defined to meet electric harness durability requirements.

**Figure 1:**
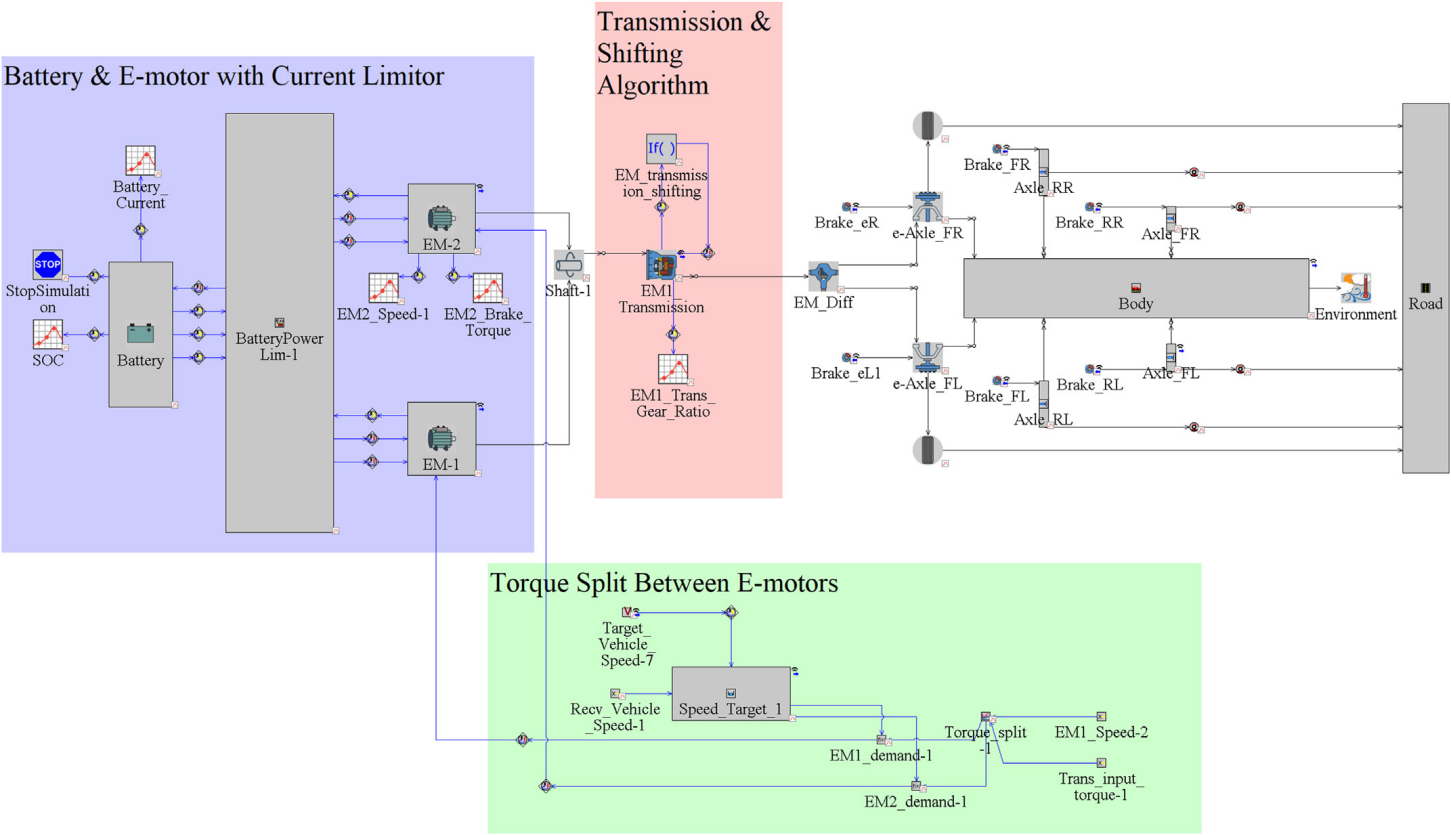
E-Truck 1D model.

Figure 1 shows the e-truck one-dimensional (1D) model generated in this study for 3-speed single transmission, and it is modified per transmission variant to replicate each powertrain architecture.

1D Battery Electric Truck model main parts are battery, e-motors, transmission, split torque algorithm, regenerative braking algorithm, and vehicle components.

**Figure 2:**
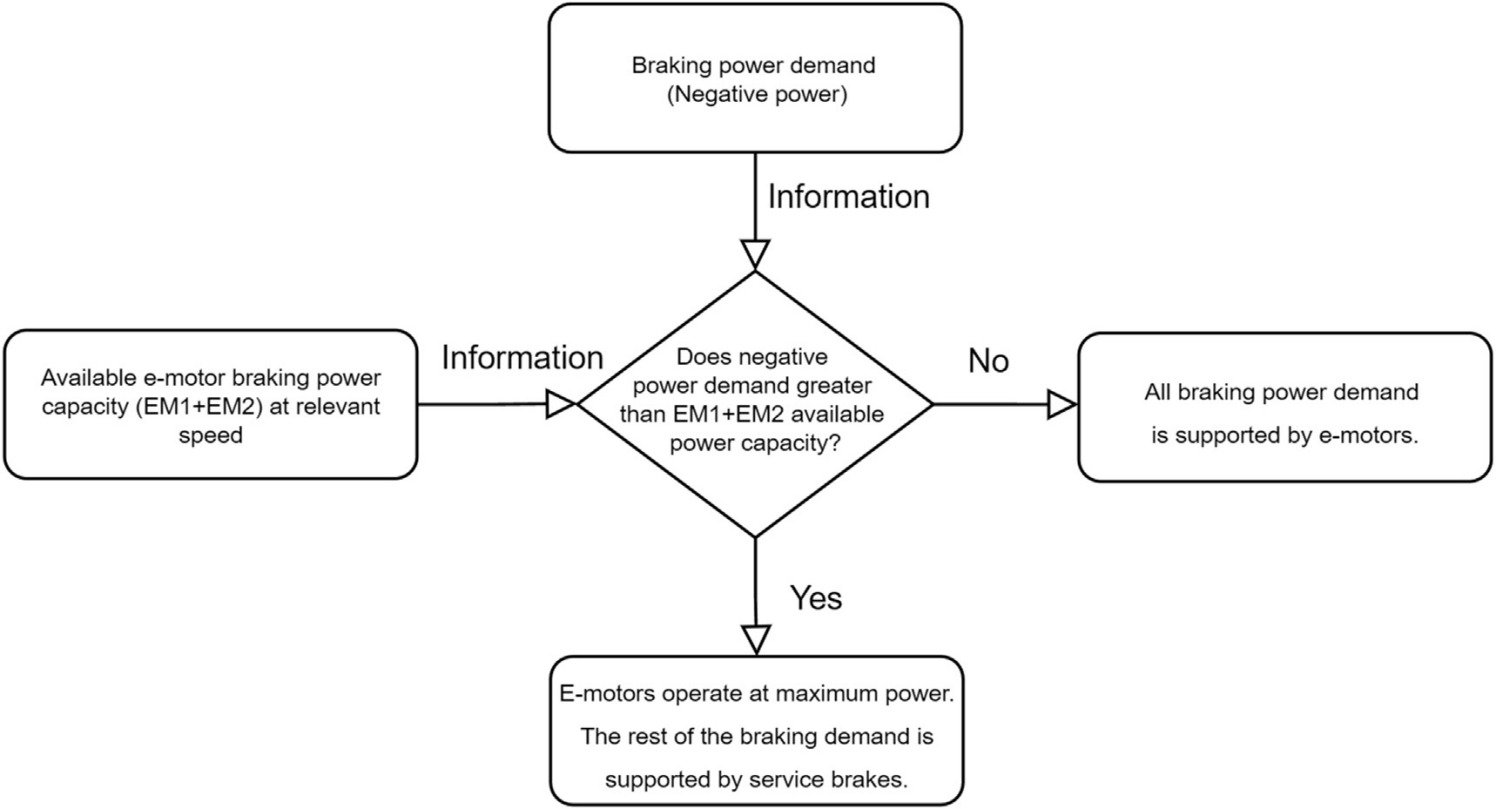
Regenerative braking algorithm.

Recuperation [24] of drive cycle energy has the highest contribution to lowering energy consumption. E-motor max power (continuous) capacity is used with the algorithm in Figure 2 to achieve the highest recuperation energy. This algorithm is using available maximum e-motor recuperation power capacity. The rest of the braking demand is handled by service brakes.

## 3 E-truck performance requirements

### 3.1 E-truck specification

E-truck vehicle specification used in one dimensional model simulation is listed in Table 1.

**Table 1:** Vehicle specification.

E-truck specification	Parameter	Value	Unit
Vehicle mass	*m*_*F*_	27 (Roadtruck)	tons
Range	-	200	km
Tyre radius	r	0.522	m
Rolling resistance coefficient	*f*_*R*_	0.005	-
Drag coefficient	*c*_*W*_	0.54	-
Vehicle frontal area	A	10	m2
Air density	*ρ*_*L*_	1.2	kg/m^3^
Gravitational acceleration	g	-	-
Gradient angle	*αSt*	Various per cycle	-
Battery cell capacity	-	37	Ah
Battery total capacity	-	240	kWh
Battery initial SOC	-	90	%
Battery charging current limitation	-	-400	A
Battery discharging current limitation	-	400	A
E-motor maximum power @ continuous	-	160 × 2	kW
E-motor maximum power @ peak	-	320 × 2	kW
E-motor positive torque limitation	-	Continuous torque limit per speed	-
E-motor negative torque limitation	-	Continuous torque limit per speed	-
E-motor working boundary	-	Continuous torque limit	-

Dual e-motors are used in this study to reach the maximum (max) target grade with continuous operation limits. The same e-motors are selected. The torque limit of e-motors is considered for continuous operation. Peak torque limits are restricted with time to protect e-motors. Since the cooling system is not finalized at the early stage of the project, peak torque operating conditions are not examined.

240 kWh battery capacity which is carried over from another BE Road Truck project, is selected for the 200 km target range. Energy consumption numbers are calculated based on consumed energy and the difference between initial SOC and final SOC at the end of the cycle.

### 3.2 Road cycle

The optimization of a powertrain can not proceed without defining the road cycles. In this study, Vecto [4] cycles are considered for energy consumption simulations.

As of 2019, the CO2 emissions and fuel consumption data determined with Vecto [5], together with other related parameters, will be monitored and reported to the Commission and made publicly available for each of those new trucks. Five different mission profiles for trucks and five different mission profiles for buses and coaches have been developed and implemented in the tool to better reflect the current European fleet.

27 tons of road truck powertrain system is optimized based on the following selected three Vecto cycles:

1. Vecto Regional Delivery Cycle (Hub to city delivery representative)
2. Vecto Urban Delivery Cycle (In city distribution truck representative)
3. Vecto Municipal Utility Cycle (Refuse truck representative)

**Figure 3:**
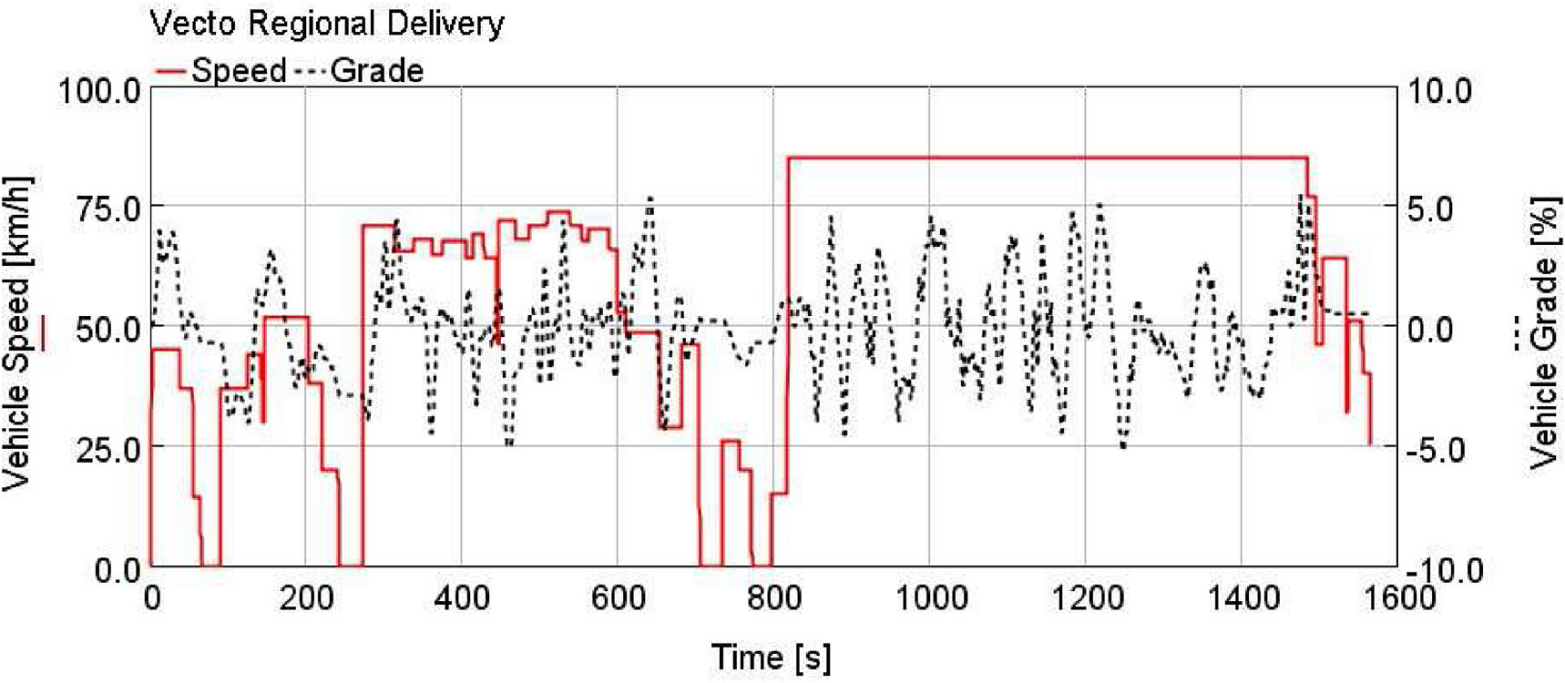
Vecto regional delivery (vecto RD).

**Figure 4:**
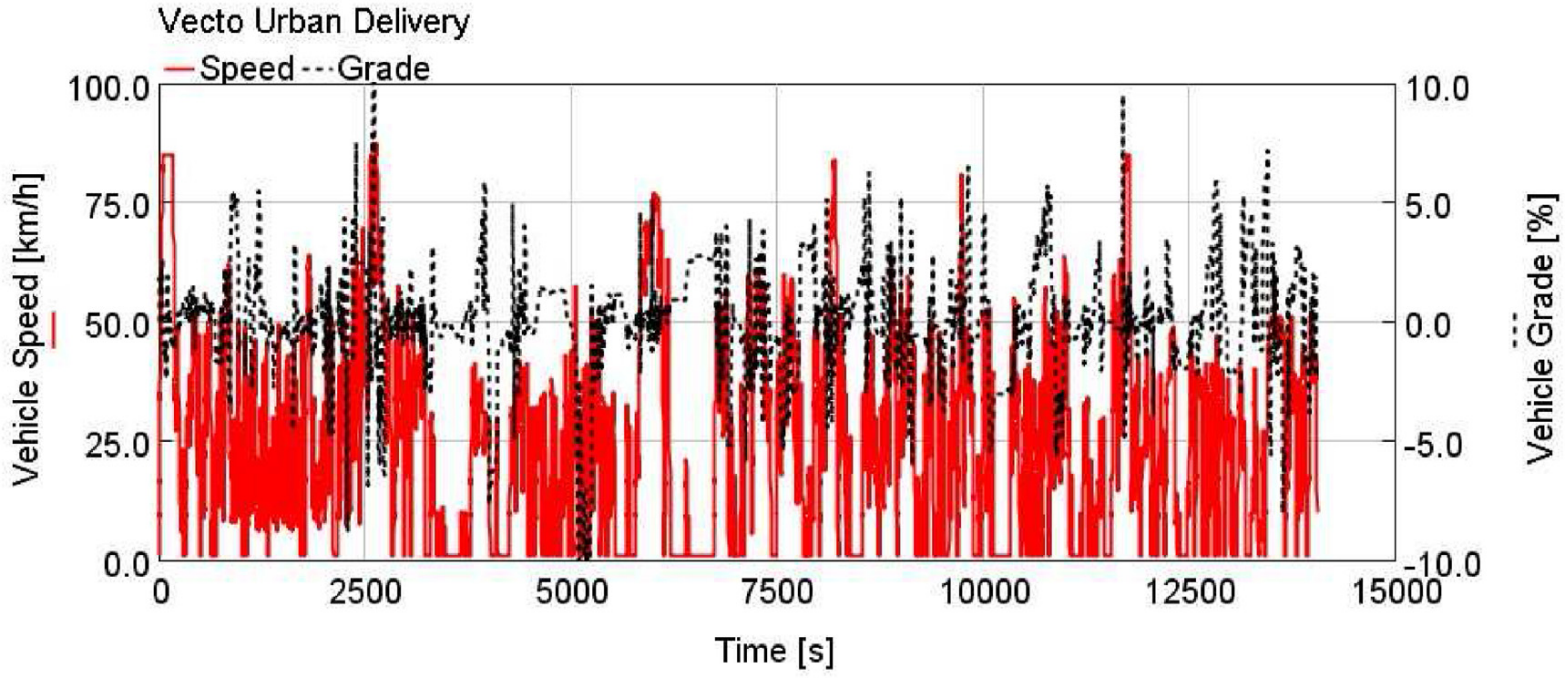
Vecto urban delivery (vecto UD).

**Figure 5:**
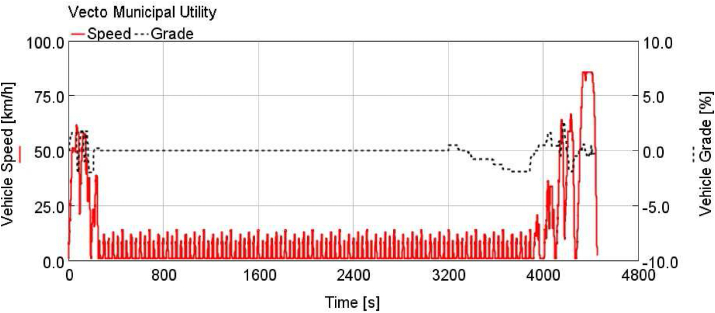
Vecto municipal utility (vecto MU).

Vehicle speeds and road grades of Vecto cycles are shown in Figures 3, 4, and 5.

### 3.3 Performance requirements

The gradeability is a crucial factor in establishing powertrain performance targets and managing the torque transfer from a power source to wheels.

In this study, the gradeability requirement of 27 tons of conventional road truck is considered to achieve the same performance.

**Figure 6:**
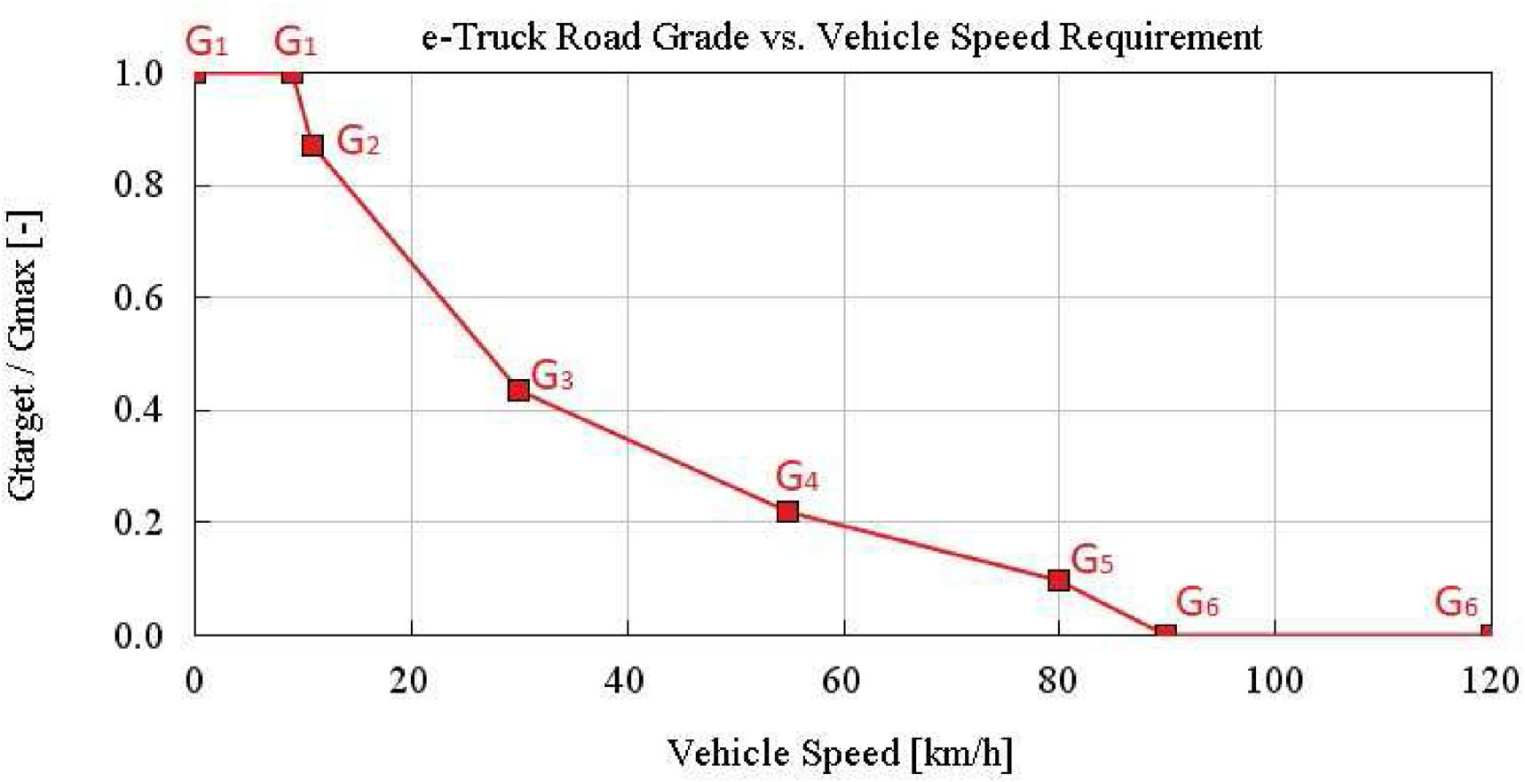
e-Truck road grade vs. vehicle speed requirement.

Figure 6 shows e-truck gradeability targets per vehicle speed. Target grades (*G*_*target*_) per vehicle speed are normalized based on the maximum grade (*G*_*max*_). *G*_1_ is the highest grade requirement, hence it is considered as *G*_*max*_.

E-truck driveline (transmission + differential) gear ratio sets are defined after conversion of grades to wheel torques per speed. The required wheel torque calculations [25] are made by Eqs. (1), (2) and (3) [25, 26].

(1) $$F=F_{R}+F_{St}+F_{L}+F_{a}$$

where:

F= Required traction force.

*F*_*R*_ = Wheel resistance force.

*F*_*St*_ = Gradient resistance force.

*F*_*L*_ = Air resistance force.

*F*_*a*_ = Acceleration resistance force.

(2) $$F=m_{F}g\left(f_{R}\;\cos\;\alpha_{St}+\;\sin\;\alpha_{St}\right)+0.5\rho_{L}c_{W}Av^{2}+m_{F}a$$

where:

F= Required traction force.

v = Vehicle speed.

a = Acceleration.

(3) T=Fr

where:

F= Required traction force.

T = Wheel torque.

The required minimum (min) and maximum (max) gear ratios (GR) of driveline per vehicle speed and grade are calculated based on maximum available continuous total e-motor torque as described in Eq. (4). In this study, provided gear ratio numbers are normalized, as shown in Eq. (5).

(4) T=Temotorμdrivelinei

where:

T = Total wheel torque.

i = Total driveline ratio (transmission + differential).

*T*_*emotor*_ = Total available torque of e-motors.

*μ*_*driveline*_ = Driveline efficiency (transmission + differential).

(5) $$i_{\mathrm{normalized}}\text{ ​}=\text{ ​}i/i_{\max}$$

where:

*i*_*normalized*_ = Normalized driveline ratio.

*i*_*max*_ = Max driveline ratio used in the analysis.

In order to meet the gradeability targets in Figure 6, minimum one normalized gear ratio of electrified powertrain driveline should be;

• Higher than 0.635 to achieve *G*_1_% grade,
• Lower than 0.411 to achieve 55 km/h in *G*_4_% grade,
• Higher than 0.141 to achieve 80 km/h in *G*_5_% grade,
• Lower than 0.188 to achieve 120 km/h in *G*_6_% grade.

## 4 Alternative transmission configurations

2-speed, 3-speed, 4-speed automated mechanical transmissions with barrel cam and follower type shifting mechanisms are studied. Single and split transmissions support driveline configurations in this study.

The total torque demand is divided between EM1 (e-motor1) powered and EM2 (e-motor2) powered drivelines. The aim is to decrease energy consumption by running EMs together in the most efficient range or shut down one of them depending on the total torque demand.

### 4.1 Single transmission and torque share

**Figure 7:**
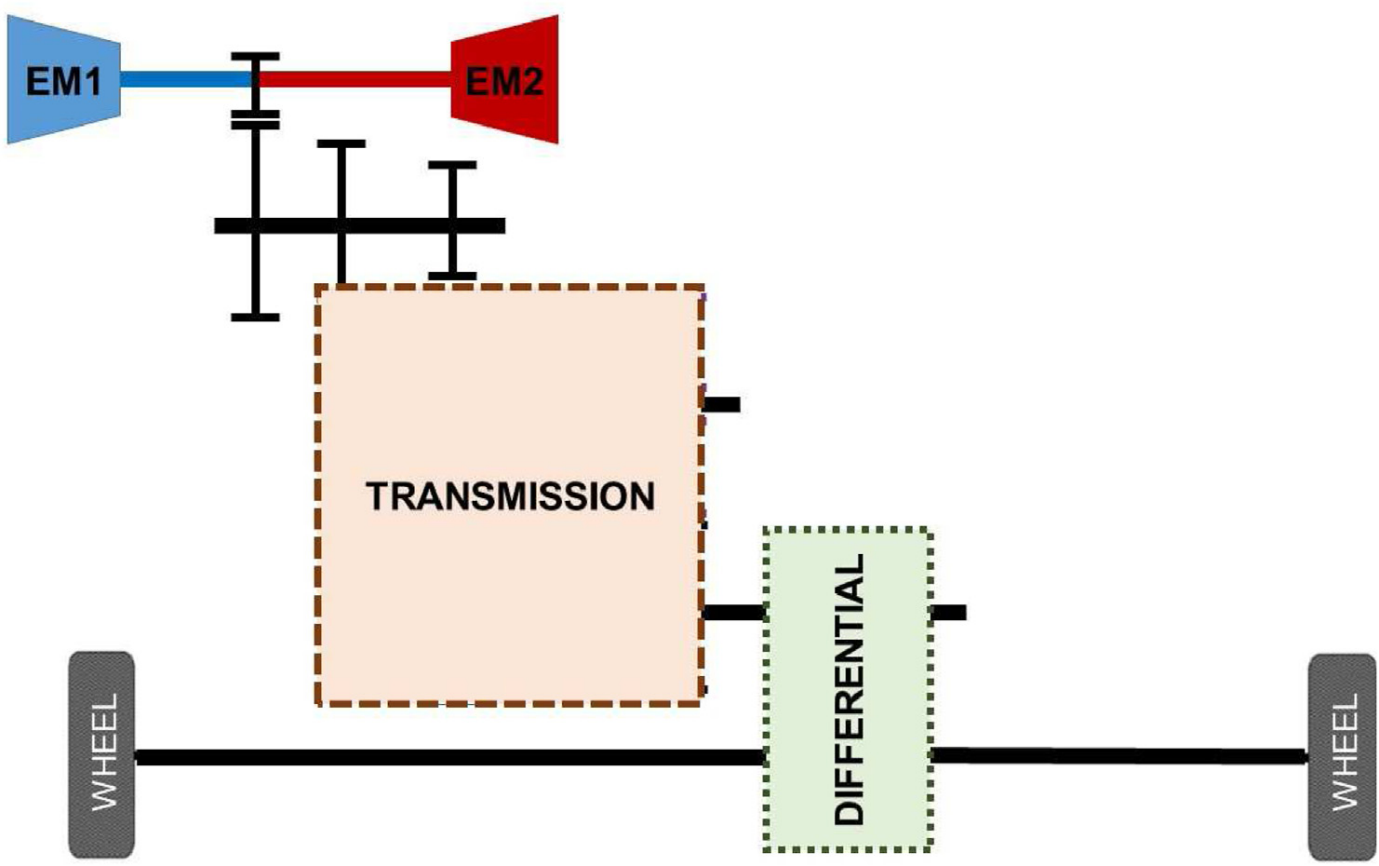
Single transmission schematic.

**Figure 8:**
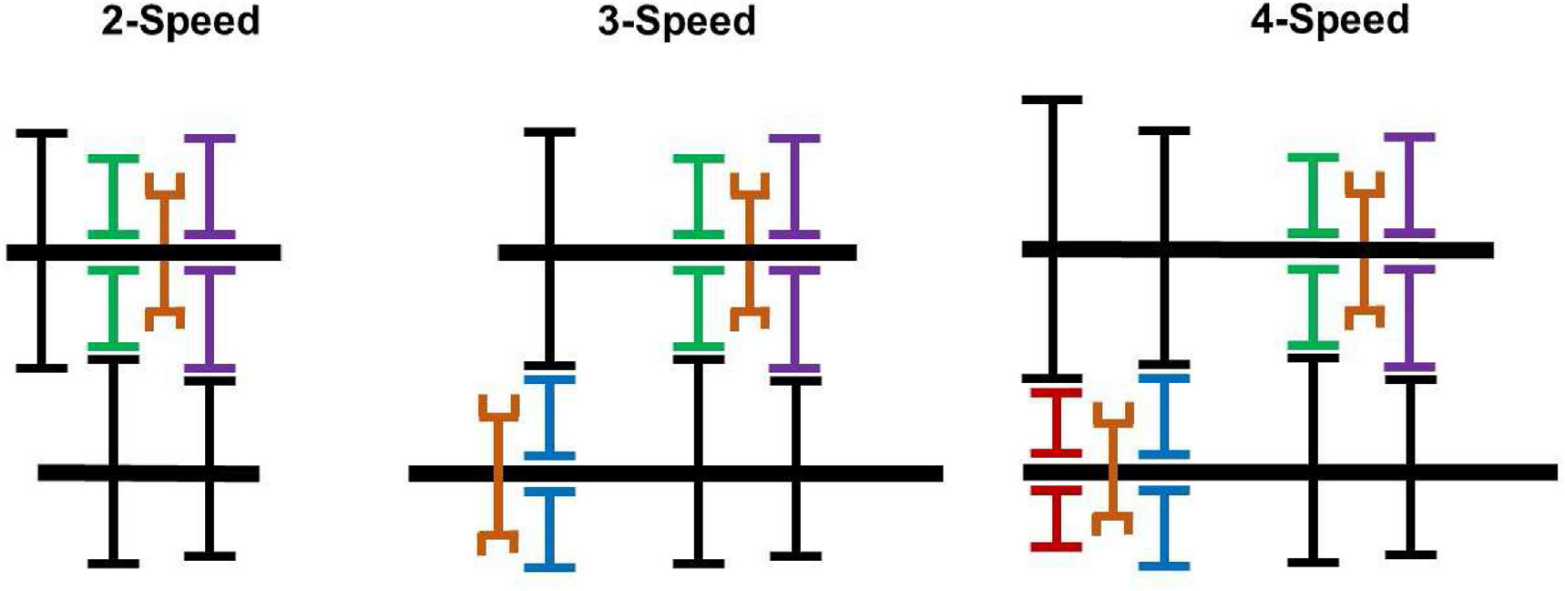
Single transmission speed alternatives.

Two e-motors are connected to a single shaft at the upstream of the transmission junction in a single transmission. It could be designed as a single-speed, or higher speeds depend on needs. This study reviews 2-speeds, 3-speeds, and 4-speeds of single transmissions, as shown in Figure 7 and Figure 8.

Torque share percent between EM1 and EM2 is studied for the single transmission.

**Figure 9:**
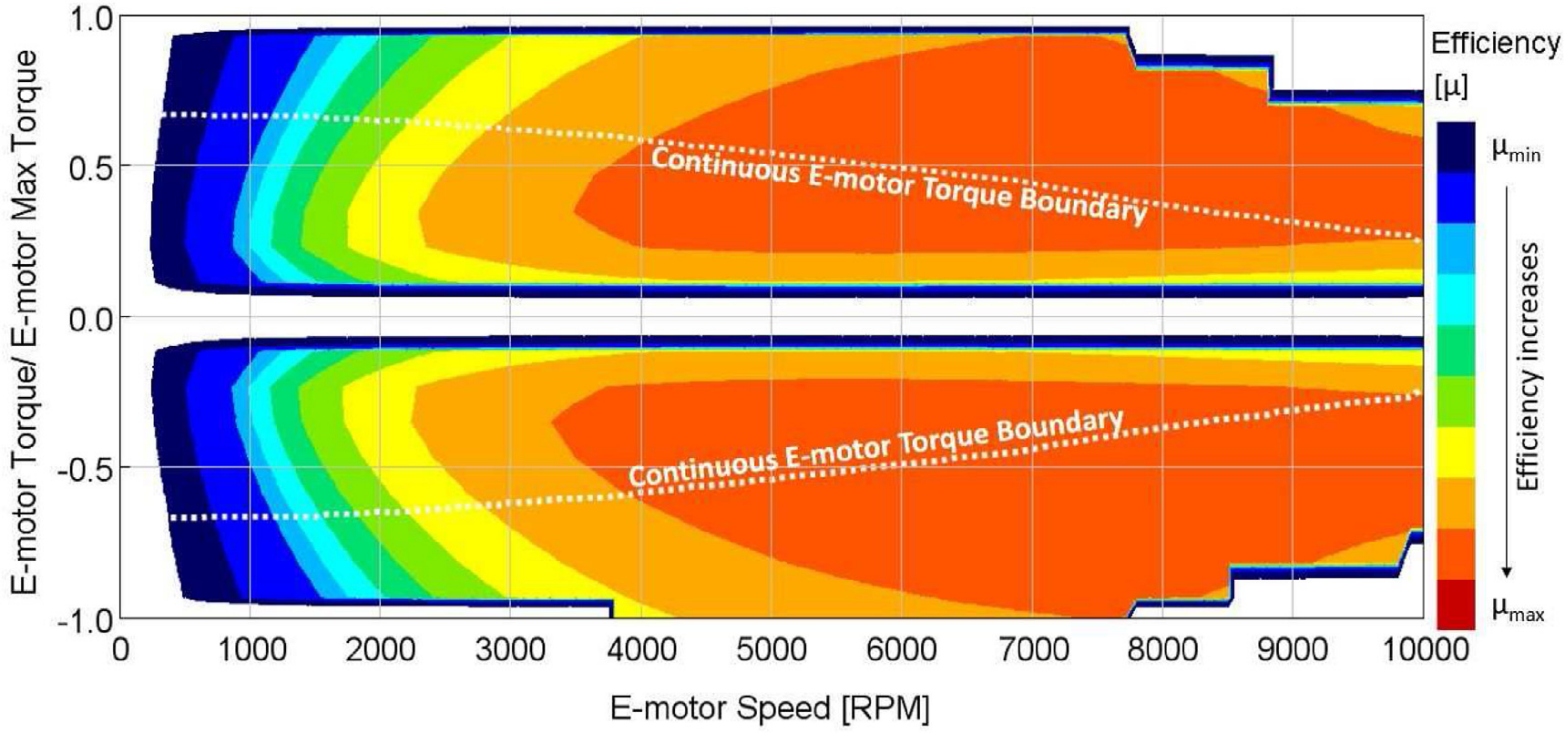
Electric motor map.

The e-motor runs in a highly efficient range between 4000-10000 RPM engine speed, as shown in Figure 9. Inside this range, efficiency is approximately 92%. Hence, 9500 and 3500 RPM values are selected as upshift and downshift speeds that maximize the e-motor operating region in a highly efficient range.

**Figure 10:**
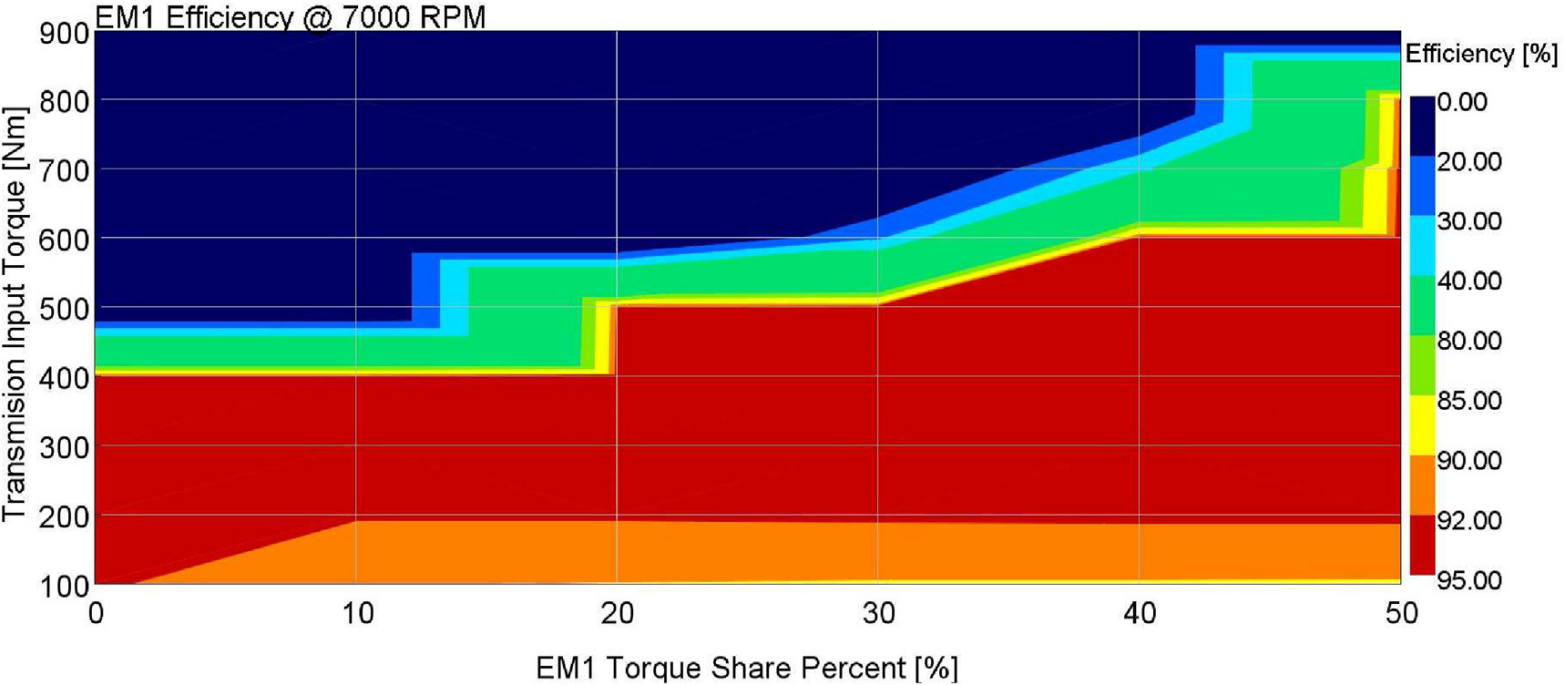
EM1 torque share split at 7000 RPM.

The EM torque share is analyzed for 900 Nm-(-900) Nm transmission input torque values, 010000 RPM e-motor speeds, 0-10-20-30-40-50% torque demand share between EM1 and EM2. E-motor efficiencies are calculated from the e-motor efficiency map for all combinations. The calculation at 7000 RPM engine speed is shown in Figure 10.

**Figure 11:**
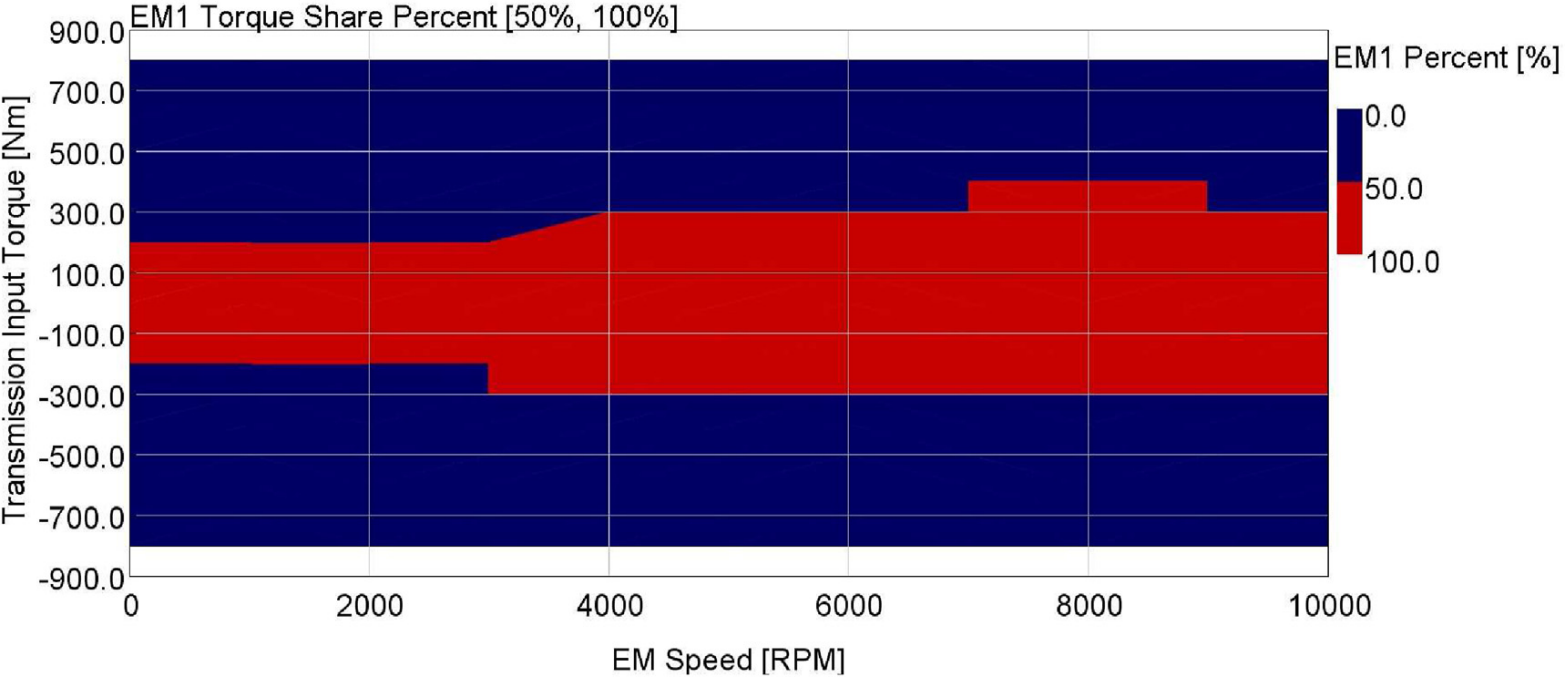
EM1 torque share percent.

This approach is completed for 0–10000 RPM with 1000 RPM increments. Afterward, the final weighted average efficiency is placed in relevant cells. The EM1 percents that provide maximum efficiency per operating conditions are shown in the 2D Map Figure 11 as a summary.

2D map of EM1 torque share percent is shown in Figure 11. The red area shows 100% EM1 torque support. This area represents shut down condition for EM2 for lower energy consumption since EM1 can support the entire torque demand with high efficient operation. EM2 percent is equal to (100-EM1).

### 4.2 Dual E-motors and its benefits

The main motivation of dual (160kW∗2) e-motors instead of one-single (320 kW) e-motor can be listed as:

1. Using the off the shelf product for lower cost values.
2. To gain an advantage in terms of power consumption by turning off one of the e-motors during operation.
3. Using split transmission’s torque fill feature in gear changes.

The simulation results show that, for total e-motor torque demands below 200 Nm, running one e-motor and turning off the other provides an advantage in terms of total energy consumption. Although this advantage varies on a cycle basis, it is an essential item that should be considered for maximizing the vehicle range.

The simulation results using double and single e-motor for 3-speed transmission are shown in Table 2.

**Table 2:** Comparison of dual E-motor energy consumption.

Route Single	320kW e-motor	Dual 160kW∗2 e-motor	Energy Saving Benefit
-	kWh	kWh	%
Vecto RD	33.63	33.62	0.05
Vecto UD	124.78	124.45	0.26
Vecto MU	13.58	13.49	0.66

As it is demonstrated in the results, increasing the duration of cycle-based low load demand increases the gained energy from turning off one of the e-motors in dual motor application.

### 4.3 Split transmission and torque share

**Figure 12:**
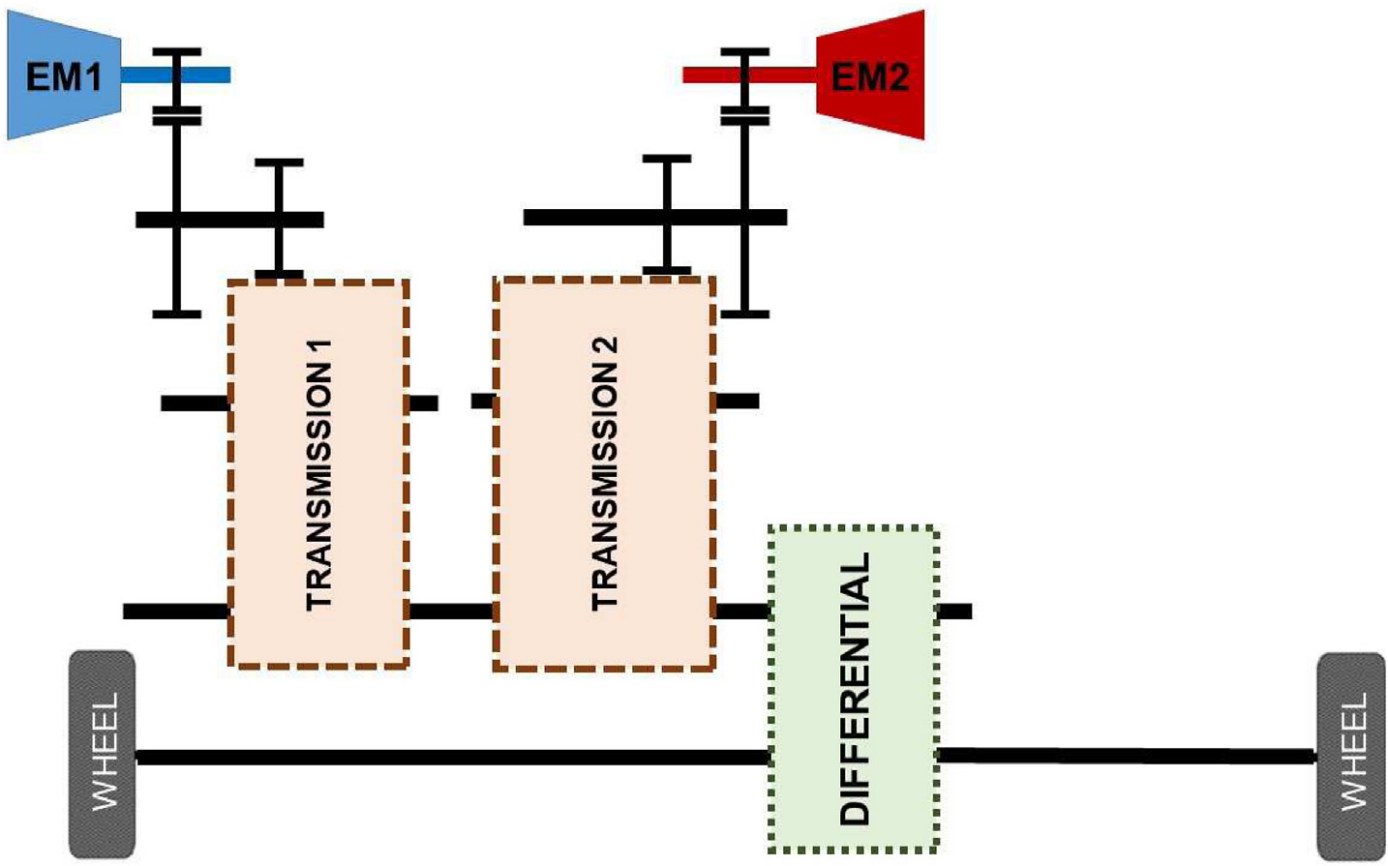
Split transmission schematic.

**Figure 13:**
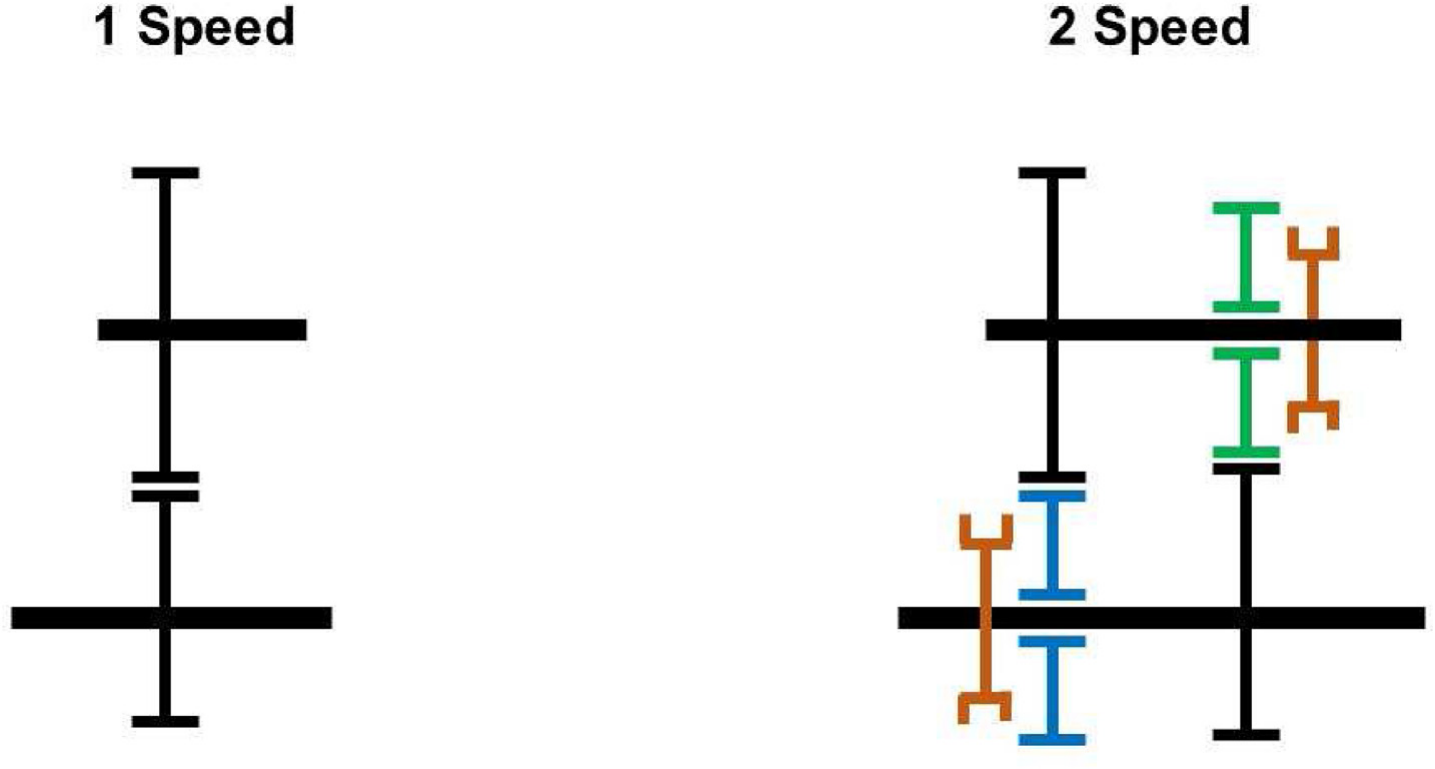
Split transmission speed alternatives.

Two e-motors are separately connected to the transmission in split transmission systems in which the total torque demand splits between drivelines. They could run drivelines in the same or different speeds from each other. In this study 2 + 1 speed and 2 + 2 speed applications are examined. Figure 12 and Figure 13 shows architecture of these applications. 2 + 1 speed split transmission indicates that one e-motor has 2-speed control and the other one has single speed control. In addition, 2 + 2 speed split transmission indicates both e-motors have 2-speed control.

This type of transmission has torque fill benefit [27] during upshift and downshift conditions. Hence, the split transmission ensures higher customer satisfaction thanks to improvement in driveability.

The torque share between EM1 and EM2 is perplexing condition in split transmission since the e-motors operate at different speeds due to different gear ratios. In order to solve this problem, an online torque split algorithm is established.

**Figure 14:**
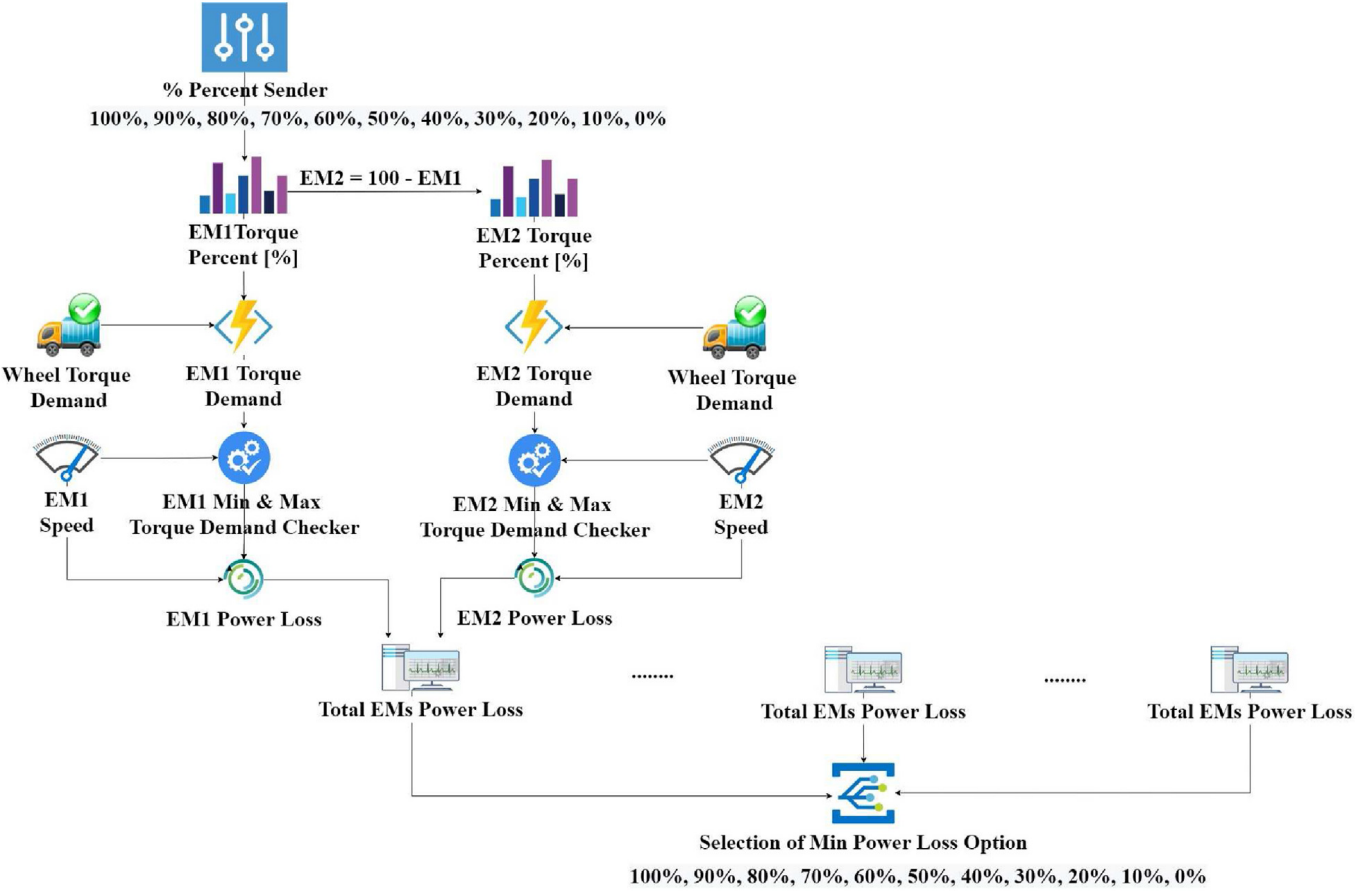
Online EM torque split algoritm.

This method checks possibility of 100%–0% percent torque share between EM1 and EM2 and related total energy consumption. Afterward, it finds the torque share percent that provides minimum energy consumption at every second of the simulation. This method’s main advantage is to shut down one EM to save battery energy during the cycle. Furthermore, it can minimize the energy consumption by checking all possible torque share percentages at every single second. The online torque split algorithm for complex transmissions is shown in Figure 14.

### 4.4 Gear shifting consistency

Tian [8] focused on the gearshift control logic of a two-speed transmission for BEVs. This study provides a valuable reference for gear shifting control of clutchless automated manual transmission in battery electric vehicles. The gear ratio of the transmission is constant during the driving and braking operations. The Motor Control Unit (MCU) manages to meet total tractive torque demands and controls the power inverter to achieve driver inputs.

Definition of the upshift and downshift speeds are critical to maximize overall e-motor efficiency. E-motor runs in a highly efficient range between 4000-10000 RPM, as shown in Figure 9. Inside this range, efficiency is approximately 92%.

9500 and 3500 RPMs are selected as upshift and downshift speeds that keep the e-motor operating points at highly efficient region of the map. After selection of upshift and downshift speeds, it is very critical to set *i*_*n,n*−1_ correctly in order to eliminate hysteresis during shifting. The ratio of *i*_*n,n*−1*,max*_ defines the maximum possible ratio between sequential gears in as shown in Eqs. (6) and (7).

(6) $${\text{i}}_{n,n-1}={\text{i}}_{n/in-1}$$

where:

*i*_*n,n*−1_ = The ratio of sequential gears.

*i*_*n*_ = The gear ratio of n-speed.

*i*_*n*−1_ = The gear ratio of (n-1)-speed.

(7) $$i_{n,n-1,\max}<S_{upshift}/S_{downshift}$$

where:

*i*_*n,n*_−_1*,max*_ = The maximum ratio of sequential gears.

*S*_*upshift*_ = Upshift speed of transmission.

*S*_*downshift*_ = Downshift speed of transmission.

Exceeding *i*_*n,n*−1*,max*_ ratio causes hysteresis (gear hunting) between gear shifts [26, 28]. Therefore, increasing the transmission speed number provides an opportunity to decrease the *i*_*n,n*−1*,max*_ ratio and keep the e-motor in a higher efficient range.

## 5 Results

This section aims to find out the optimum transmission speed and gear ratio numbers [29] that meet gradeability requirements with minimum energy consumption [30] per transmission alternatives.

### 5.1 Optimization setup

The first step of transmission optimization is defining the first and last speed gear ratios (GR). The minimum limit of the first gear ratio comes from the maximum standing grade. The maximum limit of the last gear ratio comes from the maximum target speed. Other limitations are calculated from the requirements of target vehicle speed per grade. All maximum and minimum normalized gear ratio numbers are used to define limits of optimization boundary.

Design of experiment (DOE) range gear ratio limits for all single-speed transmissions (2-speed, 3-speed, 4-speed) are shown in Table 3. The selected upshift and downshift speeds, detailed in.

**Table 3:** Single transmission DOE range.

	2-speed	3-speed	4 speed
Downshift Speed	2500	3500	5000
Upshift Speed	9500	9500	8500
Min GR of 1st speed	0.635	0.635	0.635
Max GR of 1st speed	1	1	1
Min GR of 2nd speed	0.141	0.235	0.412
Max GR of 2nd speed	0.188	0.412	0.635
Max ratio of *i*_2*,*1_	3.8	2.71	1.70
Min GR of 3rd speed	x	0.141	0.188
Max GR of 3rd speed	x	0.188	0.412
Max ratio of *i*_3*,*2_	x	2.71	1.70
Min GR of 4th speed	x	X	0.141
Max GR of 4th speed	x	X	0.188
Max ratio of *i*_4*,*3_	x	X	1.70

Section 4.1 are also listed in the table. Provided gear ratio numbers are normalized values per Eq. (5) in Section 3.3. Maximum sequential gear ratios are calculated as *i*_2*,*1_, *i*_3*,*2_, and *i*_4*,*3_ according to Eq. (7). After defining the DOE range, these values are entered into the model and the energy consumption values for all variants are examined.

The DOE range limits of all split transmissions are shown in Table 4. The selection of the GR limits of split transmission is approached in more detail, since different torque sharing of the drivelines is possible. The minimum gear ratio limit of first speeds come from the maximum standing grade.

**Table 4:** Split transmission DOE range.

	2 + 1 speed	2 + 2 speed
Downshift Speed	2500	2500
Upshift Speed	9500	9500
Min GR of 1st speed	*i*_11_ + *i*_21_*≥*1.27	*i*_11_ + *i*_21_*≥*1.27
Max GR of 1st speed	0.741	0.741
Min GR of 2nd speed	*i*_12_ + *i*_22_*≥*0.258	*i*_12_ + *i*_22_*≥*0.258
Max GR of 2nd speed	0.188	0.188
Max ratio of *i*_2*,*1_	*<*3.8	*<*3.8

Since EM1 and EM2 drivelines can operate at different speeds, the sum of first gear ratios should be greater than 1.27. The maximum limit of the last gear ratios come from the top target speed. Therefore, the gear ratio of last speeds should be less than 0.188 to achieve max speed. This condition is not possible for 2 + 1 speed transmission since one gear can not be greater than 0.635 and less than 0.188, concurrently. The other limitations are arisen from the target vehicle speed per grade requirements.

Gear ratios in the split transmission are shown as below:

1. *i*_11_ indicates the EM1 driveline and 1st speed gear ratio
2. *i*_12_ indicates the EM1 driveline and 2nd speed gear ratio
3. *i*_21_ indicates the EM2 driveline and 1st speed gear ratio
4. *i*_22_ indicates the EM2 driveline and 2nd speed gear ratio

The one-dimensional simulations of all transmission alternatives are performed for three different Vecto Cyles, given in Section 3.2. The energy consumption results are gathered from the model according to the final battery state of charge. The gear ratio sets that provide the lowest energy consumption values are selected as best case per transmission type.

### 5.2 Results and discussion

2-speed single, 3-speed single, 4-speed single, 2 + 1 speed split, and 2 + 2 speed split transmission designs are investigated based on following two criteria:

1. one dimensional e-truck model battery energy consumption results
2. gradeability checks per wheel torque curve in comparison with requirement

All transmission alternatives’ best gear ratios that achieve the highest energy savings are shown in Table 5. Results are shared as energy consumption comparison with single 2-speed transmission’s best case. The positive percentage values represent the lower energy consumption, and the negative percentage values demonstrate the higher energy consumption compared to the 2-speed single transmission.

**Table 5:** Energy consumption comparison of transmission options.

	Option1	Option2	Option3	Option4
Transmission Type	Single	Single	Split	Split
Transmission Speed	2-speed	3-speed	2 + 2 speed	2 + 2 speed
Torque Split of E-motors	50-50 split	Full variable	Full variable	Full variable
Transmission GR Set1	0.635–0.188	0.776–0.365-0.153	0.705–0.188	0.624–0.188
Transmission GR Set2	-	-	0.565–0.148	0.376–0.188
EM Torque Boundary	Continuous	Continuous	Continuous	Peak
Gradeability	Not Capable	Capable	Capable	Capable
Energy Consumption of VRD [%]	Base	-1.1	-7.6	-6.9
Energy Consumption of VUD [%]	Base	9.1	2.5	6.4
Energy Consumption of VMU [%]	Base	5.1	-1.3	0

**Figure 15:**
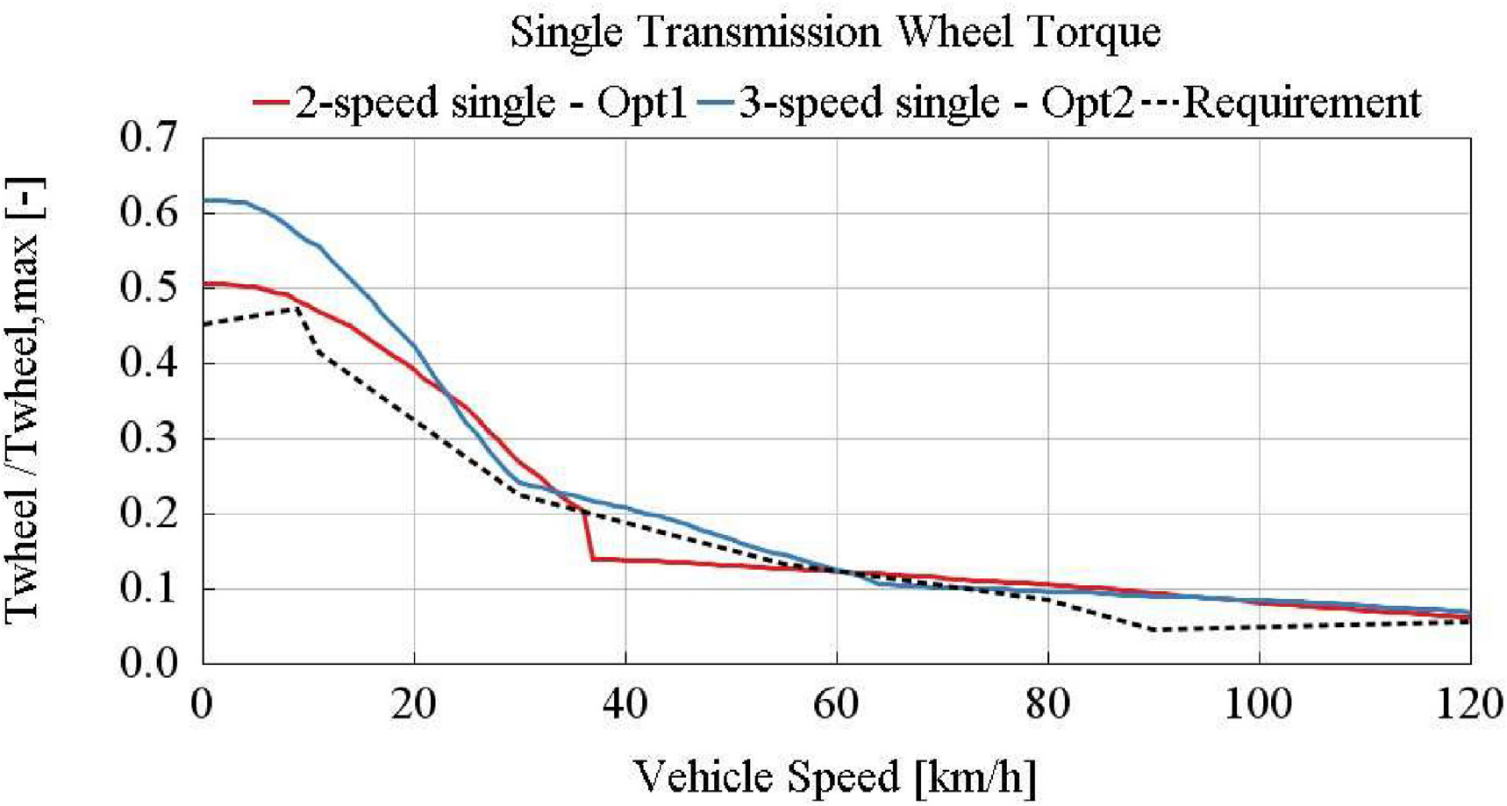
Single transmission best cases - option 1 and 2.

In Figure 15, normalized wheel torques of option 1 and 2, and e-truck requirement are shown. The 3-speed single transmission can fulfill the gradeability requirements as it is almost above entire requirement line. 2-speed transmission can not satisfy all of the requirements, as it is below requirement in 35–60 km/h vehicle speed range.

2 + 1 speed split transmission can not meet gradeability and max speed requirements at the same time. The usage of 2-speed transmission with a single e-motor is not capable of fulfilling the max speed or max grade requirements. Hence minimum 2-speed conversion is required for each e-motor. Depending on gear ratio selection, one of the three conditions (max grade, max vehicle speed, mid vehicle speeds with given grades) lays outside the boundary.

**Figure 16:**
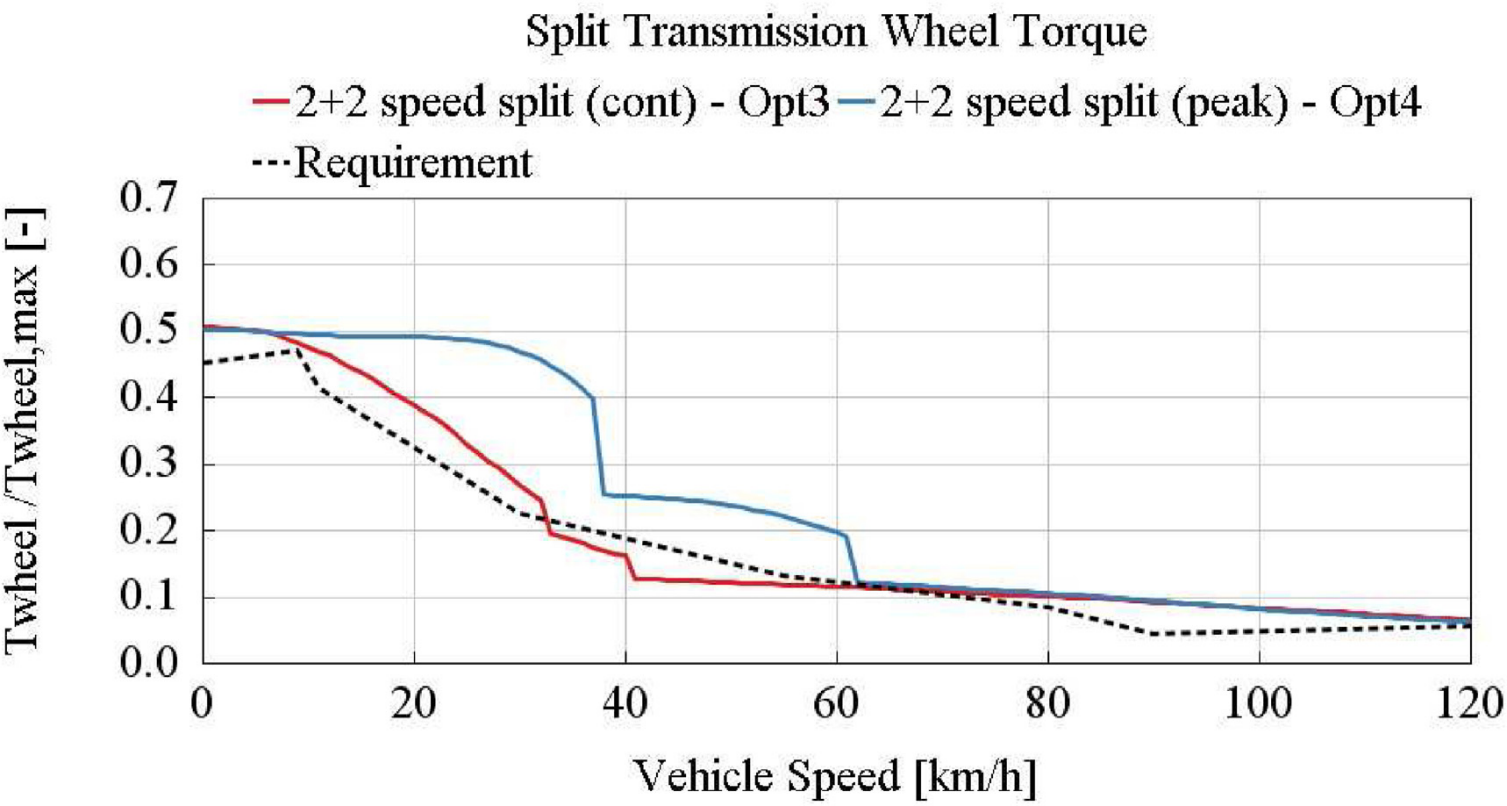
Split transmission best cases - option 3 and 4.

In Figure 16, normalized wheel torques of 2 + 2 speed split transmission best cases (option 3 and 4), and e-truck requirement are shown. The e-motor’s continuous tractive power boundary can not be filled entirely with its gear ratio combinations. It includes out-of service areas (range below wheel torque requirement) where the available power can not be used due to the selected ratios.

**Figure 17:**
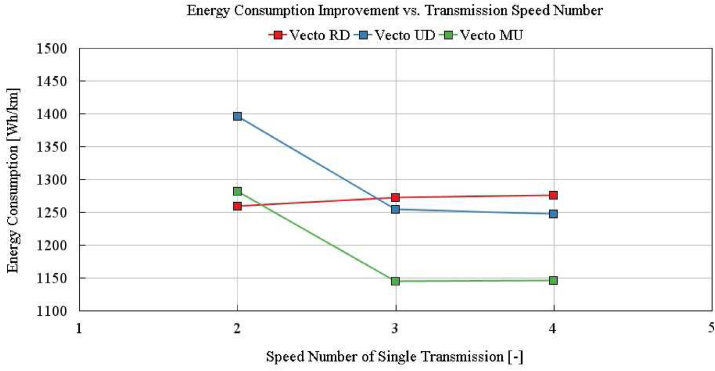
Single transmission speed optimization.

According to the battery energy consumption results of the one dimensional model in Table 5 and gradeability checks in Figures 15 and 16, best cases per transmission alternatives are selected. 2-speed single (Opt1), 3-speed single (Opt2), 4-speed single, 2 + 1 speed split, and 2 + 2 speed split (Opt3 and 4) transmission variants are investigated and the following conclusions can be drawn:

1. 2-speed single transmission is not capable of achieving the given gradeability targets as shown in Figure 15. The minimum of the three different gear ratios (maximum grade, maximum vehicle speed, medium vehicle speeds with given grades) are required.
2. 3-speed single transmission can satisfy the gradeability requirements, as shown in Figure 15. It provides the lowest energy consumption compared with 2-speed single, 2 + 1 split, 2 + 2 split transmissions.
3. 2 + 1 speed split transmission can not meet gradeability and maximum speed requirements. When only one-speed is defined for one of the e-motor, it is not possible to achieve all of the vehicle target speed-grade. Furthermore, required wheel torques per target speed-grade can only be achieved when both e-motors support at the same time in this application. Hence minimum 2-speed conversion is required for each e-motor. Depending on gear ratio selection, one of three conditions (maximum target grade, maximum target vehicle speed, medium vehicle speeds with requested grades) stays outside the boundary.
4. The 2 + 2 speed split transmission with fully variable torque split provides close results to the 3-speed single transmission. The wheel torque curve is shown in Figure 16. However, it can not provide a fully capable design within the e-motor continuous operation boundary, as shown in Figure 16. Even though the e-motor peak torque is turned on, the 2 + 2 speed split transmission can not achieve to the 3-speed single transmission energy consumption efficiency. Nevertheless, the 2 + 2 speed split transmission can provide additional 3.9% higher energy consumption benefit than continuous operation in the Vecto Urban Delivery cycle with turning on peak torques (0–10 km/h).
5. For total e-motor torque demands below 200Nm, running one e-motor and turning off the other one has an advantage in terms of total energy consumption as detailed in Section 4.2. Although the amount of this advantage varies on a cycle basis as shown in Table 2, control of dual e-motors is an essential item that should be taken into consideration in maximizing the vehicle range. Increasing the duration of cycle-based low load demand increases the gained energy from turning off one of the e-motors in dual motor applications.
6. 4-speed single transmission provides promising results as a 3-speed single transmission. According to the detailed DOE analysis, 4-speed single transmission energy consumption benefit over 3-speed single transmission is not above 0.5% overall. The energy consumption of single transmission per speed number is shown in Figure 17. Improvement above 3-speed in the single transmission is not that significant.

## 6 Conclusion

The energy consumption reduction decreases power demand from BE Truck batteries. Although lower energy consumption ensures higher efficiency and truck range, decreasing energy consumption is not the only design parameter since the performance-based customer expectations remain the same with a conventional truck. Therefore, lower energy consumption with high performance is a crucial target for BE Trucks. As a result, the performance metrics are defined with certain vehicle speeds at specific grades.

In this paper, the methodology used for BE Truck powertrain optimization is developed in three main steps.

In the first step, the vehicle performance requirements and targets are defined. These limitations are critical due to their significant effect on minimum & maximum gear ratios for the transmission design.

Secondly, the electrified powertrain model setup is created in the one-dimensional modeling environment, and a regenerative braking control algorithm is implemented. In the third step, alternative transmission configurations are studied with dual e-motor controls. EM torque sharing algorithms are defined as “50/50 torque split” and “fully variable (online) torque split” in this study. In order to achieve minimum energy consumption, “50/50 torque split” is used in single transmission designs and “fully variable torque split” in split transmission designs.

A wide range of transmission speed and gear ratio sets are defined for all transmission alternatives to optimize energy consumption and fulfill gradeability-based requirements. Energy consumption comparison of five alternative transmission designs (2-speed single, 2 + 1 speed split, 2 + 2 speed split, 3-speed single, and 4-speed single) are accomplished.

It is also proven that the applications using two e-motors of the same speed&power range require similar transmission speeds to get higher total e-motor efficiency. It is an expected condition since their optimum speeds, which provide lower energy consumption, are the same. On the other hand, increasing the number of gearbox speeds from three to four could only bring 0.5 % extra energy consumption benefit as shown in Figure 17. This result suggests that the energy consumption benefit of using an infinite number of gears would not be significant.

After taking the points listed above into consideration, it is critical to note that; although the transmission speed and ratio optimization results are valid for a specific application, the unique methodology developed within this paper is applicable for different applications.

## Declarations

### Author contribution statement

Elif Gözen: Conceived and designed the experiments; Performed the experiments; Analyzed and interpreted the data; Contributed reagents, materials, analysis tools or data; Wrote the paper.

Emre Özgül: Conceived and designed the experiments; Analyzed and interpreted the data; Wrote the paper.

M. Sedat Çevirgen: Analyzed and interpreted the data; Contributed reagents, materials, analysis tools or data; Wrote the paper.

### Funding statement

This work was supported by the European Union’s Horizon 2020 research and innovation programme under Grant Agreement no. 874972.

### Data availability statement

The data that has been used is confidential.

### Declaration of interest’s statement

The authors declare no conflict of interest.

### Additional information

No additional information is available for this paper.
